# How Are Special Interest Sessions Used by Higher Psychiatry Trainees in Wessex Deanery?

**DOI:** 10.1192/bjo.2022.125

**Published:** 2022-06-20

**Authors:** Claire Gee

**Affiliations:** Southern Health NHS Foundation Trust, Southampton, United Kingdom

## Abstract

**Aims:**

The aim of this survey was to establish how higher psychiatry trainees are using special interest sessions. Special interest sessions provide trainees with the opportunity to gain additional experience and are defined as ‘a clinical or clinically related area of service which cannot be provided within the training post but which is of direct relevance to the prospective career pathway of the trainee.’ The Curriculum for Specialist Training in Psychiatry states that two sessions every week must be devoted during each year of Specialty training for such personal development, which may be taken in research or to pursue special clinical interests.

**Methods:**

All higher psychiatry trainees working within Health Education England (HEE) Wessex Deanery were invited to complete a survey using Google Forms between 1^st^ March 2021 and 1^st^ April 2021. The survey included multiple choice and open questions relating to the accessibility, use and content of special interest sessions. Participants were asked to comment on their experiences. Quantitative data were analysed using Excel and qualitative data were collated and reviewed.

**Results:**

20 of the total 42 higher psychiatry trainees responded with the highest response rates from trainees in Old Age Psychiatry and dual training posts. 25% were using all their entitled special interest sessions. The remaining trainees were not able to use them consistently due to clinical service demands and 10% were not using any due to being unaware of opportunities available.

The majority of trainees were using special interest sessions for research, followed by postgraduate qualifications and psychotherapy. Other special interests included medical education, management experience and psychiatric liaison. 70% found their special interest sessions straightforward to arrange and supervisors were highlighted as a useful support.

Most trainees did not have a good awareness of special interest opportunities available within their specialty. 90% would like to be better informed of opportunities for special interest sessions.

**Conclusion:**

The survey indicated that the majority of higher psychiatry trainees are having difficulty accessing special interest sessions due to clinical service demands and a lack of awareness of opportunities available. In order to meet Curriculum requirements, it is important that trainees are supported by supervisors and trusts to access special interest sessions. Specialty training job descriptions should allow for special interest time with appropriate cover arrangements. To improve awareness of special interest sessions, I have developed an information booklet listing opportunities available for higher psychiatry trainees in HEE Wessex Deanery.

